# EGCG Prevents High Fat Diet-Induced Changes in Gut Microbiota, Decreases of DNA Strand Breaks, and Changes in Expression and DNA Methylation of* Dnmt1* and* MLH1* in C57BL/6J Male Mice

**DOI:** 10.1155/2017/3079148

**Published:** 2017-01-04

**Authors:** Marlene Remely, Franziska Ferk, Sonja Sterneder, Tahereh Setayesh, Sylvia Roth, Tatjana Kepcija, Rahil Noorizadeh, Irene Rebhan, Martina Greunz, Johanna Beckmann, Karl-Heinz Wagner, Siegfried Knasmüller, Alexander G. Haslberger

**Affiliations:** ^1^Department of Nutritional Sciences, University of Vienna, Vienna, Austria; ^2^Institute of Cancer Research, Department of Medicine I, Medical University of Vienna, Vienna, Austria

## Abstract

Obesity as a multifactorial disorder involves low-grade inflammation, increased reactive oxygen species incidence, gut microbiota aberrations, and epigenetic consequences. Thus, prevention and therapies with epigenetic active antioxidants, (−)-Epigallocatechin-3-gallate (EGCG), are of increasing interest. DNA damage, DNA methylation and gene expression of* DNA methyltransferase 1*,* interleukin 6*, and* MutL homologue 1* were analyzed in C57BL/6J male mice fed a high-fat diet (HFD) or a control diet (CD) with and without EGCG supplementation. Gut microbiota was analyzed with quantitative real-time polymerase chain reaction. An induction of DNA damage was observed, as a consequence of HFD-feeding, whereas EGCG supplementation decreased DNA damage. HFD-feeding induced a higher inflammatory status. Supplementation reversed these effects, resulting in tissue specific gene expression and methylation patterns of* DNA methyltransferase 1* and* MutL homologue 1*. HFD feeding caused a significant lower bacterial abundance. The* Firmicutes/Bacteroidetes* ratio is significantly lower in HFD + EGCG but higher in CD + EGCG compared to control groups. The results demonstrate the impact of EGCG on the one hand on gut microbiota which together with dietary components affects host health. On the other hand effects may derive from antioxidative activities as well as epigenetic modifications observed on CpG methylation but also likely to include other epigenetic elements.

## 1. Introduction

Metabolic syndrome, a multifactorial disorder, results from a long-term imbalance of diet and physical activity, genetic predisposition, and an imbalanced gut microbiota influencing several metabolic pathways including epigenetic regulation. In 2015, the prevalence of metabolic syndrome was determined at 1.9 billion adults being overweight (BMI ≥ 25 kg/m^2^ (body mass index)) and more than 600 billion obese (BMI ≥ 30 kg/m^2^) [1]. Thus, this high incidence of obesity and associated diseases like type 2 diabetes (hyperglycemia on basis of an insulin resistance) are a challenge and financial burden for national health care systems.

Increased adipose tissue is an important energy storage but also a key endocrine organ with active metabolism relevant in energy homeostasis, lipid and glucose metabolism, fibrinolysis, coagulation, blood pressure, and inflammation (like interleukin-6 (IL-6), tumor necrosis factor *α* (TNF-*α*)) [2, 3], but also in increased reactive oxygen species (ROS) and free fatty acids (FFAs) production, and increased oxidative stress [4, 5]. Oxidative stress in turn is associated with both genome-wide hypomethylation and promoter hypermethylation of the DNA [6], resulting in transcriptional silencing of key antioxidant enzymes as well as tumor suppressor genes [7, 8]. Diets rich in bioactive anti-inflammatory compounds, such as polyphenols, have been recommended to reduce oxidative stress and to decrease inflammation [9].

EGCG ((−)-Epigallocatechin-3-gallate), the main catechin of green tea (50–75%), has been shown to support many potential health effects, including antioxidant, anticarcinogenic, hypocholesterolemic, and cardioprotective epigenetic activities [10]. It was found to increase energy expenditure and weight loss, reduce fat mass, and facilitate weight maintenance after weight loss [11]. Antiobesity effects might be mediated through antioxidative and singlet oxygen quencher properties. Inhibition of destructive effects of ROS might act through selective inhibition of specific enzyme activities such as (*Dnmt*s) DNA methyltransferases, repair of chromosomal aberrations [12], and suppressing inflammation in the development of obesity [9]. EGCG supplementation (0.1%) in obese and diabetic C57BL/KsJ-db/db mice decreased the levels of insulin, IGF-1 (insulin-like growth factors), IGF-2, free fatty acid, and expression of* TNF-α*, interleukin-* (IL-) 6*,* IL-1β*, and* IL-18* mRNAs in hepatic tissue [13]. EGCG inhibits* Dnmt* activity resulting in a decreased 5-methylcytosine concentration; 20 *μ*mol/L of EGCG already inhibited* Dnmt* activity in oesophageal (KYSE-150), colon (HT-29), prostate (PC-3), and breast (MCF7 and MDA-MB-231) cancer cells, although no effects of EGCG on* Dnmt* activity (2–50 *μ*mol/L) are also shown in cancer cells. Another* Dnmt* inhibitory pathway of flavan-3-ols results from an increase of S-adenosyl-l-homocysteine (SAH) [14]. A reduced* Dnmt* activity reactivates methylation-silenced genes in a dose (5–50 *μ*mM of EGCG) and time dependent (12–144 h) manner [25].

According to Fang et al. (2003)* MutL homologue 1 (MLH1)* and* O6-methylguanine-deoxyribonucleic acid methyltransferase (MGMT)*, both part of the DNA mismatch repair (MMR) system, are hypomethylated in the CpG rich promoter region [25] accompanied by a higher expression of mRNA [25]. In contrast, ROS-induced oxidative stress contributes to hypermethylation of normally unmethylated promoter regions, resulting in transcriptional silencing of key antioxidant enzymes as well as tumor suppressor genes [7, 8]. EGCG also alters histone acetyl transferase (HAT) activity, resulting in aberrant chromatin structures, as well as miRNA expression in hepatocellular carcinoma cells [14].

However, above small-intestinal absorption, tea catechins could reach the large intestine and be processed by gut microbiota into gallic acid and epigallocatechin (EGC) [26, 27]. Alterations in gut microbiota composition and differences in gut microbial metabolite profile due to different dietary feeding offer insights that may be relevant for several chronic conditions, including obesity. The obesity related gut microbiota is composed by a less bacterial diversity and altered abundance, gene-representation, and metabolic pathways [28]. These differences include a higher abundance of* Firmicutes*, whereas* Bacteroidetes* are reduced resulting in a higher* Firmicutes/Bacteroidetes* ratio in obese individuals. An increased abundance of* Lactobacilli* is mentioned as a growth promoter and is associated with weight gain and inflammatory processes during obesity [29]. Genes encoding for carbohydrate metabolism enzymes are increased in the gut microbiome of obese mice, provoking a greater capacity to extract energy from the diet and to generate short-chain fatty acids (SCFAs) [30, 31]. SCFAs are essential for the microbial community and play a role in regulation of energy balance, inflammatory processes, health, and obesity [32].

Meanwhile interventions with EGCG resulted in a reduction of* Clostridium *spp. abundance, increased* Bacteroides*, but also influenced* Bifidobacterium* and* Prevotella,* to a lesser extent resulting in lower levels of acetic and butyric acids and little influence on propionic acid levels in caecum. Thus, effects on weight reduction and weight maintenance due to dietary intervention with EGCG could be responsible for the regulation of energy metabolism in the body [26].

In the present study, we investigated the effects of a physiologically applicable dose of EGCG on gut microbiota, DNA damage, DNA methylation, and gene expression of inflammatory mediators:* IL-6; Dnmt1*, and DNA repair:* MLH1* in liver and colon due to metabolic syndrome induced by a high fat diet (HFD) and due to control diet (CD) with a focus on associations between alteration of DNA repair processes, immune functions, and genomic instability. We investigated the colon as direct nutrient contact is given, but also hepatic tissue, the main organ in glucose and insulin metabolism. In addition, gut microbiota composition and diversity were analyzed on the basis of stool samples. Effects of dietary EGCG in alleviating conditions associated with obesity and metabolic syndromes are reported.

## 2. Materials and Methods

The Ethical Committee of the Medical University of Vienna approved the animal experiment (BMWFW-66.009/0329-WF/V/3b/2014) implemented with 6-week-old C57BL/6J male mice (*n* = 60) (Janvier Labs, France). Three animals were kept per cage (Macrolon type III, Techniplast GmbH, Germany) under standard conditions (24 ± 1°C, humidity 50 ± 5%, 12 hrs light/dark cycle). The animals were divided into four groups after an acclimatization time of two weeks with control diet (EF R/M control, 12% fat ssniff Spezialdiäten GmbH, Soest, Germany): (1) CD group (EF R/M control, 11% kJ fat, ssniff Spezialdiäten GmbH, Soest, Germany), (2) CD plus EGCG group (CD + EGCG) (EGCG: 25 mg/kg body weight per day), (3) HFD group (54% kJ fat ssniff EF acc.D12492 (I) mod., ssniff Spezialdiäten GmbH, Soest, Germany), and (4) a HFD plus EGCG group (HFD + EGCG). Food and water were provided ad libitum and supplemented with EGCG in the intervention groups (25 mg/kg b.w.). EGCG was provided as pure EGCG (EGCG-Uji-XP, System Biologie, Wollerau). Water/EGCG uptake was measured daily. Once per week the mice were weighted and food intake was determined (Table 1). After 4 months, the animals were killed by cervical dislocation.

### 2.1. Single-Cell Gel Electrophoresis (SCGE) Assay

**Figure 1:**
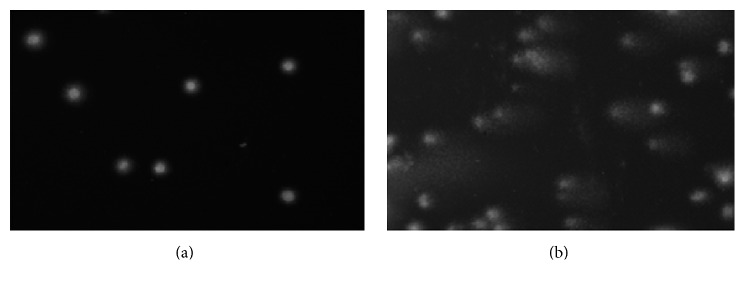
Photographic images of “comets” in an intact nucleus (a) and in a nucleus with damaged DNA (b). Stain: ethidium bromide.

DNA migration in an electric field was analyzed in hepatocytes and colonocytes from mice in SCGE assays (comet assay) [33, 34]. This approach has the advantage that measurement can be conducted with variety of organs [35] and is increasingly used in genetic toxicology Collins, (2015) [36]. A typical picture from a “comet” as marker for DNA-instability is shown in (Figure 1).

Nuclei from livers and cells from colons were collected according to the method developed by Sasaki et al. (2002) [37]. The procedure for the measurement of DNA migration in liver cells and colonocytes was employed by us in a number of earlier studies [38–42]. 1.0 g liver tissue was homogenized by use of a Potter Elvehjem-type (B. Braun, Melsungen, Germany) at 400 rpm in 4.0 mL chilled homogenization buffer (pH 7.5). Subsequently, the homogenates were centrifuged (800*g*, 10 min, 4°C). Colon cells were isolated by scratching mucosa from the colons and were kept on ice in 2.0 mL homogenization buffer. The nuclei were suspended in LMPA (0.5%, Gibco, Paisley, UK) and transferred to slides which were precoated with NMPA (1.0%, Gibco, Paisley, UK).

The experiments were carried out according to Burlinson et al. (2007) [43]. After lysis (pH 10.0) and electrophoresis (20 min, 300 mA, 25 V, at 4°C, and pH > 13), the gels were stained with ethidium bromide (20 *μ*g/mL, Sigma-Aldrich, Germany).

With nuclei from each organ, three slides were prepared per experimental point and 50 cells were evaluated from each slide. Slides were examined under a fluorescence microscope (Nikon EFD-3, Japan) using 25-fold magnification. DNA migration was determined with a computer aided comet assay image analysis system (Comet Assay IV, Perceptive Instruments, UK).

### 2.2. Gene Expression Analysis

Liver and colon samples were stored at −80°C until RNA and DNA isolation using the AllPrep DNA/RNA/miRNA Universal Kit (Qiagen, Germany) according to the manufacturer's protocol. Concentration, respectively, purity was controlled with a Picodrop100 (Picodrop, UK). Complementary DNA (cDNA) was synthesized by reverse transcription using RT_2_ First Strand Kit (Qiagen, Germany). cDNA was analyzed with real-time polymerase chain reaction (PCR) using qPCR Primer Assays (Qiagen, Germany) and RT_2_ SYBR Green Master Mix (Qiagen, Germany) according to manufacturer's protocol. PCR conditions were as follows: initial step of 95°C for 10 min, followed by 40 cycles of 95°C for 15 s and 60°C for 1 min, ending with melting curve analysis (gradient melting of the products was performed at 0.5°C/10 s from 65°C to 95°C). Each sample was analyzed in duplicate with normalization to the housekeeping gene glyceraldehyde-3-phosphate-dehydrogenase (GAPDH).

### 2.3. Methylation Analysis

Genomic DNA was bisulfite converted with EpiTect® Fast Bisulfite Conversion kit (Qiagen, Germany) and amplified by PCR using the PyroMark PCR Kit (Qiagen, Germany) according to manufacturer's instructions with primers for* Dnmt1* and* MLH1* designed by PyroMark Assay Design SW 2.0 Software (Table 2).

The PCR was carried out in a total reaction volume of 25.0 *μ*L, containing 12.5 *μ*L PyroMark 2x PCR master mix, 10 pmol (*Dnmt1*) or 7 pmol (*MLH1*) of each primer, 2.5 *μ*L CoralLoad Concentrate 10x (Qiagen, Germany), and 10.0 ng (*Dnmt1*) or 15.0 ng (*MLH1*) bisulfite converted DNA. Thermocycling started with initial denaturation at 95°C for 15 min, followed by 45 cycles at 94°C for 30 s, 55.5°C for 45 s, 72°C for 45 s, and a final extension at 72°C for 10 min. PCR product quality was investigated with agarose gel electrophoresis. CpG methylation analysis was performed in a PyroMark Q24 MDx (Qiagen, Germany).

### 2.4. Gut Microbiota Analysis

Stool was collected before intervention and continuously after 1 month until the end of the study period and stored at −20°C until microbial DNA extraction by using the QIAamp® Fast DNA Mini Kit (Qiagen, Germany) following the manufacturer's protocol including two steps of 45 sec beadbeating at 4000 rpm with a 60 sec break in between to increase the DNA yield. DNA concentration and purification were measured using Pico100 (Picodrop Ltd., Cambridge, UK).

The abundance of gut microbial subgroups was determined by 16s rDNA using quantitative real-time PCR with SYBR green or TaqMan-Probe Master Mix with specific primer pairs (Tables 3 and 4) in a Rotor Gene 3000 (Corbett Life Science, Australia). The PCR reaction mixtures and serial DNA dilution of typical strains were prepared according to Remely et al. (2013) [44].

The diversity of gut microbiota was analyzed using denaturing gradient gel electrophoresis (DGGE) [44].

### 2.5. Statistical Analyses

For statistical analyses of SCGE assay results GraphPad Prism 5.02 (GraphPad Software, USA) was used. The means and standard deviation (SD) of % DNA in the comet tails of the nuclei from the different treatment groups were calculated. Comparisons of groups were done with Student's* t*-test based on the means of three slides/animal.

All statistical analyses of gene expression and methylation analysis were performed using IBM SPSS Advanced Statistics 20.0 (SPSS, USA). All data are shown means ± SD. ΔCT values were calculated by normalization to GAPDH (ΔCT = CT-Target − CT-GAPDH). The ΔΔCT value shows the difference between the two groups (ΔΔCT = ΔCT + EGCG −  ΔCT-Control). Relative changes in gene expression between the intervention and control group are determined by the 2^−ΔΔCT^ equation (fold change = 2^−ΔΔCT^). Kolmogorov-Smirnov-Test was used to test the normalization of the data. The Mann-Whitney *U*-Test was used to examine significant relationships. For all comparisons *p* values ≤ 0.05 were considered as statistically significant.

## 3. Results

### 3.1. Body Weight, Food Intake, and EGCG Uptake

**Figure 2:**
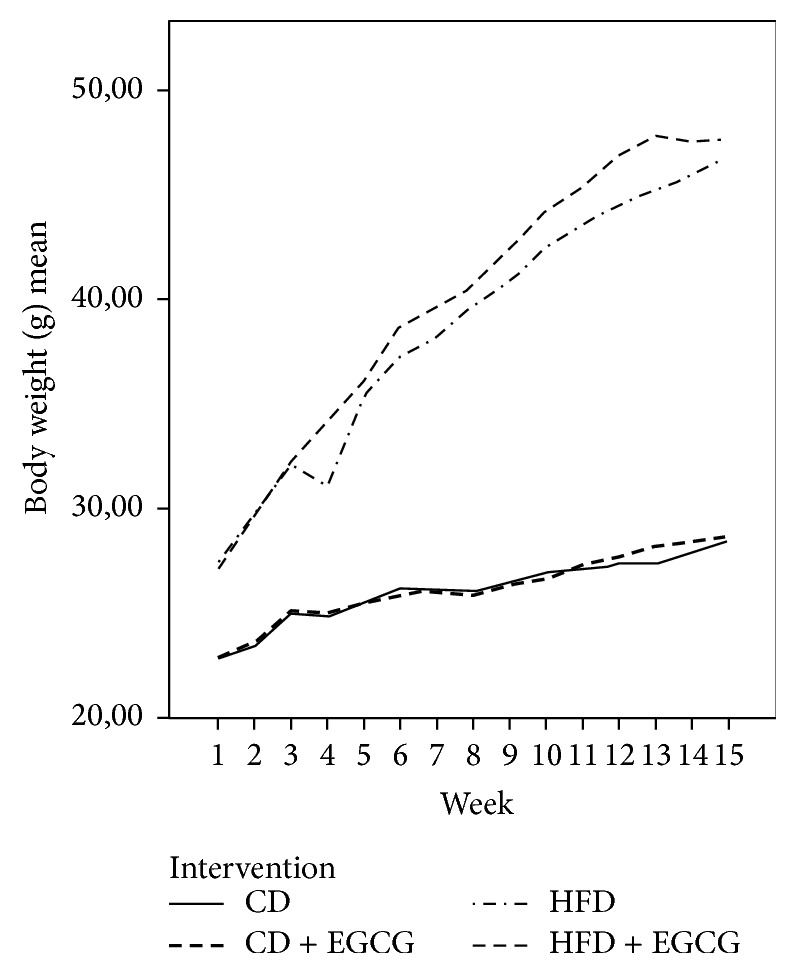
Body weight gain of C57BL/6J male mice over 4 months (CD = control diet, HFD = high fat diet, CD + EGCG = control diet plus EGCG, and HFD + EGCG = high fat diet plus EGCG).

Body weight and food intake were measured weekly, water/EGCG uptake daily. According to Table 1 food intake and total water consumption did not differ between the groups (Table 1). Mean EGCG uptake was about 0.64 ± 0.07 mg in the CD group and about 0.97 ± 0.09 mg in the HFD group of each mouse per day. Mice fed a HFD (T1: 30.54 ± 3.43 g; T4: 45.99 ± 4.46 g) and HFD + EGCG (T1: 30.74 ± 3.43 g; T4: 47.69 ± 3.45 g) increased significantly more weight in comparison to CD fed mice (T1: 23.99 ± 1.50 g; T4: 27.88 ± 1.49 g) and CD + EGCG (T1: 24.16 ± 1.52 g; T4: 28.43 ± 1.76 g) (*p* ≤ 0.01, Figure 2). The body weight increase over study period was significant in all groups (*p* ≤ 0.01).

### 3.2. SCGE Experiments with Nuclei from Colon and Liver Cells

**Figure 3:**
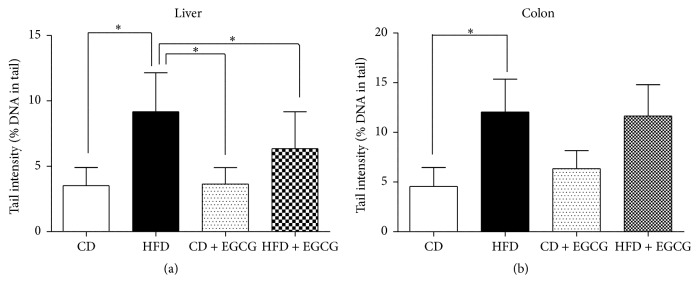
Impact of EGCG supplementation on DNA damage in liver (a) and colon (b) of C57BL/6J male mice. Bars indicate means ± SD of results obtained with 15 animals per group. From each sample, three slides were made and 50 cells were evaluated per slide (CD = control diet, HFD = high fat diet, and CD + EGCG = control diet plus EGCG; HFD + EGCG = high fat diet plus EGCG; stars indicate significance: ^*∗*^ *p* value ≤ 0.05).

HFD induced significant DNA damage in liver and colon compared with CD. In liver of animals fed with HFD and supplemented with EGCG in drinking water the extent of DNA migration was significantly decreased by 31.5% (*p* ≤ 0.05) after supplementation (Figure 3(a)). No effect was detected in colon of HFD group after EGCG supplementation Figure 3(b).

Supplementation with EGCG in CD group in colon of animals caused slight DNA damage, while no effect was detected between CD and CD + EGCG in liver.

### 3.3. Relative Gene Expression and CpG Methylation of the Promoter Region of* MLH1* in Liver and Colon Cells

**Figure 4:**
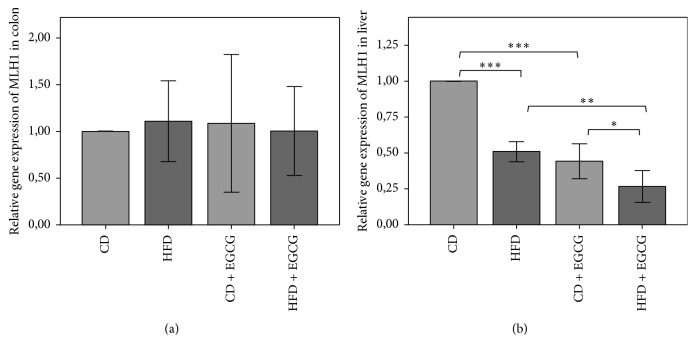
Relative gene expression of* MLH1* in colon (a) and liver (b) of C57BL/6J male mice. All gene expression data are relative to CD and were normalized to the house keeping gene* GAPDH*. Error bars represent 95% confidence intervals (CD = control diet, HFD = high fat diet, and CD + EGCG = control diet plus EGCG; HFD + EGCG = high fat diet plus EGCG; stars indicate significance: ^*∗*^ *p* value ≤ 0.05, ^*∗∗*^ *p* value ≤ 0.01, and ^*∗∗∗*^ *p* value ≤ 0.001).

In liver cells the relative gene expression of* MLH1* decreased significantly in HFD fed mice compared to the CD (49%, *p* ≤ 0.001). EGCG supplementation showed significant reduction in CD + EGCG compared to CD (56%; *p* ≤ 0.001) and in HFD + EGCG compared to HFD (44%; *p* ≤ 0.01). Moreover the protective effect by EGCG was significantly higher in HFD + EGCG than in CD + EGCG (38%; *p* ≤ 0.05) (Figure 4(b)).

The relative gene expression of* MLH1* in colon did not result in any significant difference between the interventions (Figure 4(a)).

**Figure 5:**
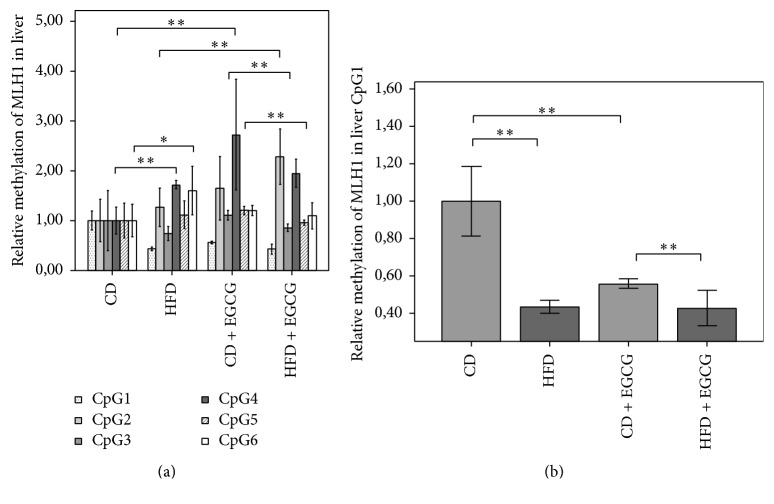
Relative CpG methylation status in promotor region of* MLH1* in liver of C57BL/6J male mice. Mean methylation data are shown for* MLH1* in each intervention group. All methylation data are relative to CD. Error bars represent 95% confidence intervals. In Figure 5(a) significance is shown for CpG 4, 5, and 6. Figure 5(b) shows significant differences in the methylation status of CpG1 (CD = control diet; HFD = high fat diet; CD + EGCG = control diet plus EGCG; HFD + EGCG = high fat diet plus EGCG; stars indicate significance: ^*∗*^ *p* value ≤ 0.05 and ^*∗∗*^ *p* value ≤ 0.01).

**Figure 6:**
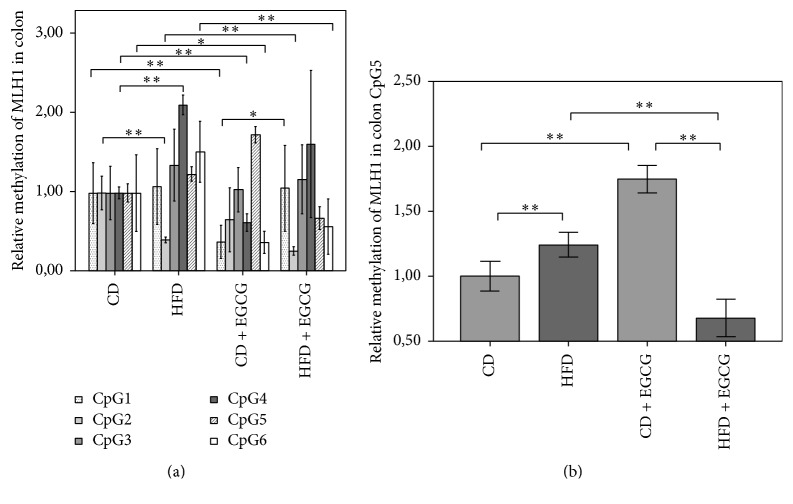
Relative CpG methylation status in promotor region of* MLH1* in colon. Mean methylation data are shown for* MLH1* as an overview (Figure 6(a)) (significant for CpG 1, 2, and 4) and CpG5 (Figure 6(b)). All methylation data are relative to CD. Error bars represent 95% confidence intervals (CD = control diet; HFD = high fat diet; CD + EGCG = control diet plus EGCG; HFD + EGCG = high fat diet plus EGCG; stars indicate significance: ^*∗*^ *p* value ≤ 0.05 and ^*∗∗*^ *p* value ≤ 0.01).

In* MLH1* promoter region the relative methylation of six CpGs was investigated in liver and in colon (Figures 5 and 6, Table 5). In liver cells CD + EGCG the mean methylation was higher compared to CD (CD: 2.83%; CD + EGCG: 3.23%) but in HFD + EGCG a decreased mean methylation status compared to HFD was shown (HFD: 3.36%; HFD + EGCG: 3.18%). In particular, at CpG1 HFD (57%) either CD + EGCG (44%) showed a significant decrease in methylation status compared to CD (*p* ≤ 0.01). Furthermore significant hypomethylation in the HFD + EGCG group was seen compared to CD + EGCG (24%; *p* ≤ 0.01; Figure 5(b)). At CpG4 (73%; *p* ≤ 0.01) and at CpG6 (60%; *p* ≤ 0.05) the methylation status significantly increased with HFD compared to CD. Hypermethylation was seen in both supplementation groups at CpG4 CD + EGCG (172%) and HFD + EGCG at CpG2 with an increase of 80% normalized to, respectively, CD or HFD (*p* ≤ 0.01). By comparison of CD + EGCG with HFD + EGCG the CD group showed significant hypomethylation at CpG3 (22%; *p* ≤ 0.01) and CpG5 (20%; *p* ≤ 0.01; Figure 5(a)).

The methylation of* MLH1* in colon cells increased with EGCG in the CD group (CD: 2.79%; CD + EGCG: 2.97%) but decreased in the HFD groups (HFD: 3.41%; HFD + EGCG: 3.01%; Figure 6). In colon cells the methylation status of* MLH1* showed a significant reduction at CpG2 in HFD compared to CD (60%; *p* ≤ 0.01). Furthermore, EGCG supplementation significantly reduced the methylation status of CpG2 in HFD + EGCG group compared to HFD (35%; *p* ≤ 0.01). A significant decrease at CpG1 was seen in CD with EGCG supplementation compared to CD (62%, *p* ≤ 0.01) whereas HFD + EGCG was significantly higher methylated compared to CD + EGCG (82%  *p* ≤ 0.05). At CpG4 HFD resulted in a hypermethylation of 113% compared to CD. Furthermore, supplementation with EGCG in the CD decreased the methylation status significantly by 38% when normalized to CD (*p* ≤ 0.01). CpG5 of* MLH1* was significantly higher methylated in HFD (24%; *p* ≤ 0.01) and in CD + EGCG (75%; *p* ≤ 0.01) compared to CD whereas in the HFD + EGCG significant hypomethylation was shown compared to CD (45%; *p* ≤ 0.01). A significant reduction was observed between CD + EGCG and HFD + EGCG animals (61%; *p* ≤ 0.01). CD + EGCG resulted in a significant hypomethylation at CpG6 of* MLH1* in colon cells compared to CD (63%; *p* ≤ 0.05) and, respectively, in HFD with (34%; *p* ≤ 0.01).

### 3.4. Relative Gene Expression of IL-6 in Colon

**Figure 7:**
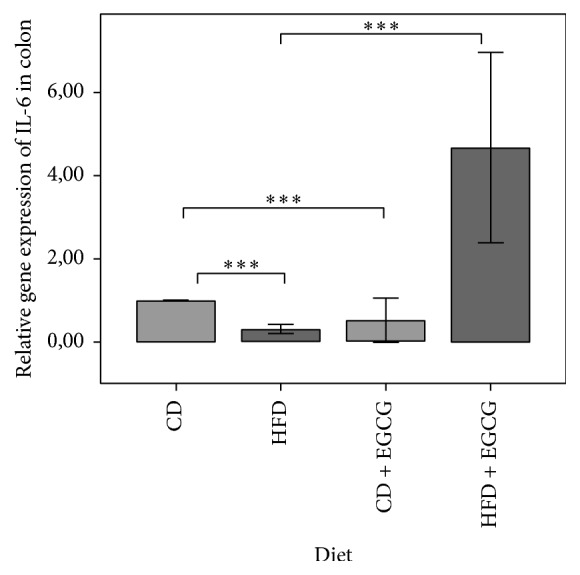
Relative gene expression of IL-6 in colon. All gene expression data are relative to CD and normalized to the house keeping gene* GAPDH*. Error bars represent 95% confidence intervals (CD = control diet; HFD = high fat diet; CD + EGCG = control diet plus EGCG; HFD + EGCG = high fat diet plus EGCG; stars indicate significance: ^*∗∗∗*^ *p* value ≤ 0.001).

A significant lower expression of IL-6 has been measured between CD and HFD animals (*p* ≤ 0.01) and also in CD and HFD + EGCG mice (*p* ≤ 0.01) in the colon (Figure 7). In liver the gene expression of IL-6 was under detection limit in the study group.

### 3.5. Relative Gene Expression and CpG Methylation of the Promoter Region of* Dnmt1* in Liver and Colon Cells

**Figure 8:**
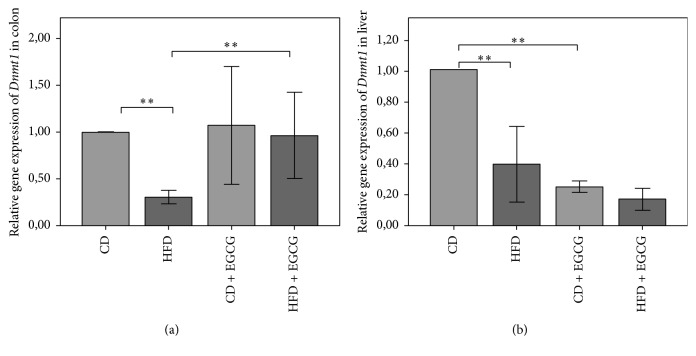
Relative gene expression of* Dnmt1* in colon (a) and liver (b) of C57BL/6J male mice. All gene expression data are relative to CD and normalized to the house keeping gene* GAPDH*. Error bars represent a 95% confidence intervals (CD = control diet; HFD = high fat diet; CD + EGCG = control diet plus EGCG; HFD + EGCG = high fat diet plus EGCG; stars indicate significance: ^*∗∗*^ *p* value ≤ 0.01).

Relative gene expression of* Dnmt1* in liver cells was lower in HFD compared to CD (61%; *p* ≤ 0.01). Supplementation with EGCG resulted in a significantly reduced gene expression compared to, respectively, CD (75%; *p* ≤ 0.01) and HFD (51%; *p* ≤ 0.01) (Figure 8(b)).

In colon the relative gene expression of* Dnmt1* decreased significantly in HFD compared to CD (69%, *p* ≤ 0.01) although the decrease of HFD compared to CD is compensated by EGCG in HFD with a more than three times higher gene expression of* Dnmt1* compared to HFD (*p* ≤ 0.01, Figure 8(a)).

**Figure 9:**
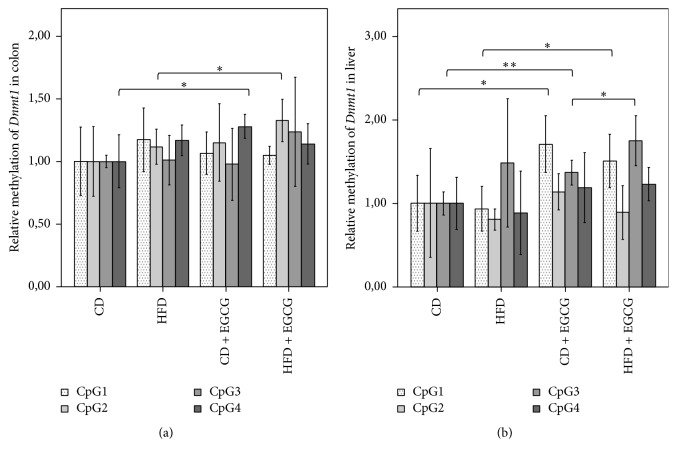
Relative CpG methylation status in promoter region of* Dnmt1* in colon (a) and liver (b) of C57BL/6J male mice. Mean methylation data are shown relative to control diet. Error bars represent a 95% confidence interval (CD = control diet; HFD = high fat diet; CD + EGCG = control diet plus EGCG; HFD + EGCG = high fat diet plus EGCG. Stars indicate significance: ^*∗*^ *p* value ≤ 0.05 and ^*∗∗*^ *p* value ≤ 0.01).

In the promoter region of* Dnmt1* in liver and colon four CpGs were analyzed (Table 5). Mean methylation of* Dnmt1* in liver cells was higher in CD + EGCG (3.21%) and HFD + EGCG (3.09%) compared to each diet CD (2.28%) and HFD (2.37%). Significant differences in methylation status of* Dnmt1* were determined in CpG1 and CpG3. In both diets EGCG significantly increased the methylation status of CpG1 (CD + EGCG: CD 71%; *p* ≤ 0.05; HFD + EGCG: HFD 62%; *p* ≤ 0.05). The same effect of EGCG was seen at CpG3 in the CD group with a significant increase (37%; *p* ≤ 0.01). Furthermore the supplementation with EGCG resulted in a significantly higher methylation of CpG3 in HFD + EGCG than in CD + EGCG (28%, *p* ≤ 0.05). CpG2 and CpG4 did not show any significant changes in methylation status (Figure 9(b)).

In colon cells CpG2 and CpG4 showed significant changes in the methylation status of* Dnmt1*. A significant higher methylation status in CpG2 was observed due to EGCG in the HFD group (19%; *p* ≤ 0.05). In CD + EGCG supplementation resulted in a significant hypermethylation at CpG4 (28%; *p* ≤ 0.05). No significant changes were observed at methylation status of* Dnmt1* at CpG1 and CpG3 (Figure 9(a)).

### 3.6. Gut Microbiota Composition and Diversity

**Figure 10:**
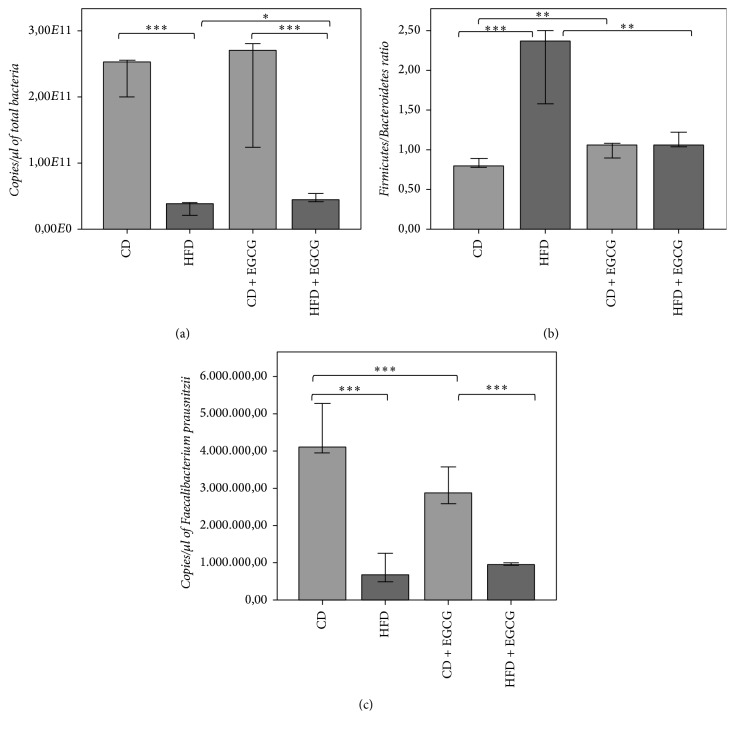
16S rDNA qPCR quantification of total bacterial abundance (a),* Firmicutes/Bacteroidetes ratio* (b), and* F. prausnitzii* (c). Error bars represent 95% confidence intervals (CD = control diet; HFD = high fat diet; CD + EGCG = control diet plus EGCG; HFD + EGCG = high fat diet plus EGCG. Stars indicate significance: ^*∗*^ *p* value ≤ 0.05, ^*∗∗*^ *p* value ≤ 0.01, and ^*∗∗∗*^ *p* value ≤ 0.001).

Differences in total bacterial abundance were shown between CD and HFD (*p* < 0.0001) and between CD + EGCG and HFD + EGCG but also between HFD and HFD + EGCG (*p* = 0.039; Figure 10(a)). HFD feeding caused a lower bacterial abundance in both HFD groups resulting in a lower microbial diversity compared to CD (HFD bands = 21.4 ± 5.08, HFD + EGCG bands = 19.6 ± 3.84, CD bands = 20.4 ± 4.62, and CD + EGCG bands = 17.02 ± 5.07).

The* Firmicutes/Bacteroidetes* ratio is significantly higher in both HFD groups compared to CD groups (*p* < 0.0001). EGCG treatment induced a significantly lower ratio in HFD + EGCG compared to HFD (*p* < 0.0001) but a significantly higher ratio in CD + EGCG compared to CD (*p* < 0.0001; Figure 10(b)).* Lactobacilli* decreased with EGCG intervention. Both clostridial clusters (*Clostridium* cluster IV,* Clostridium* cluster XIVa) were significantly lower in HFD groups compared to CD groups (*p* < 0.0001).* Clostridium cluster IV* significantly increased in HFD + EGCG compared to HFD (*p* = 0.005).* Clostridium cluster XIVa* increased in CD + EGCG compared to CD (*p* = 0.189) and was significantly more abundant in comparison to HFD + EGCG (*p* < 0.0001). In turn,* F. prausnitzii* was less abundant in HFD groups compared to CD groups (*p* < 0.0001) and was more abundant in the CD groups compared to all other groups (CD + EGCG : HFD + EGCG : *p* < 0.0001; CD : CD + EGCG: *p* = 0.001; Figure 10(c)).

In HFD mice,* butyryl CoA: acetate CoA-transferase* gene was significantly lower in comparison to CD mice (*p* < 0.0001). Intervention with EGCG in the CD group resulted in a significant decrease (*p* < 0.0001) whereas with HFD no significant effect was shown. Results of the* butyrate kinase* gene showed similar results whereas HFD + EGCG group showed a significant increase (CD : HFD *p* < 0.0001; CD : CD + EGCG *p* = 0,001; HFD : HFD + EGCG *p* = 0.005).

*Bacteroidetes* were significantly lower abundant in HFD compared to CD (*p* < 0.0001) and significantly increased with EGCG intervention in HFD mice (*p* = 0,001). An increase was shown in CD mice due to EGCG treatment.

*Akkermansia* showed a lower abundance in HFD fed mice compared to CD (*p* = 0.092). EGCG treatment resulted in a lower abundance in CD + EGCG (*p* = 0,001) but no significant change in abundance of HFD + EGCG was observable (*p* = 0.574).

## 4. Discussion

We showed that HFD induces significant DNA damage in liver and colon compared with CD. These results were also reflected by a lower gene expression of* Dnmt1* in liver and colon of HFD fed mice. In turn, DNA methylation status was higher in this group.* MLH1* methylation status was also higher compared to CD, but gene expression lower due to HFD feeding in both organs. In particular CpG1 showed a decreased methylation status in contrast to CpG4 with an increased methylation in liver. In colon CpG2, CpG4, CpG5, and CpG6 of* MLH1* promoter region were affected due to different feeding.* IL-6* gene expression was significantly higher in HFD compared to CD.

The gut microbial composition differs between CD and HFD: lower total bacterial abundance due to HFD, lower microbial diversity, higher* Firmicutes/Bacteroidetes* ratio, lower abundance of* F. prausnitzii,* and* Akkermansia,* and reduced incidence of* butyryl CoA: acetate CoA-transferase* gene and* butyrate kinase *gene.

On the basis of microbial analysis we are able to support the results of our previous publications on human fecal analysis [44]. Mice fed a HFD differ in microbial subpopulations especially the* Firmicutes/Bacteroidetes* ratio which is already handled as a marker in obesity epidemic. Although other researches also show converse or not diverging results [45–49]. However, additionally we can support results of gut microbial metabolites/cell wall components influencing the host via epigenetic mechanisms too. An increased* Firmicutes/Bacteroidetes* ratio occurs or even causes low-grade inflammation, increased* IL-6* gene expression, but also increased DNA damage and by increased* MLH1* methylation status and reduced expression of the gene. However, the aspect if gut microbial dysbiosis first induces immunological disequilibrium or genomic instability remains. Pateras et al. (2015) extensively investigated the “cross-talk” between genomic instabilities and alteration of immune functions in regard to health implications in humans [50]. Ray and Kidane (2016) indicated a microbial dysbiosis as conducive for the release of bacterial metabolites triggering chronic inflammation followed by DNA damage [51]. Karakasilioti et al. (2013) support another theory that persistent DNA damage triggers chronic inflammation in adipose tissue [52]. Inhibition of cyclooxygenase 2, implicated in the generation of prooxidant eicosanoids, resulted in a decrease of 8-isoprostane, plasma thiobarbituric acid reactive substance, and an increased GSH/GSSG (glutathione/glutathione-disulfide) ratio in rats [53]. Thus, microbial dysbiosis may induce four different mechanisms of DNA damage: (1) mitogen released by a dysbiotic microbiota possesses the capacity to enter the cell and enhance reactive oxygen and nitrogen species (RONs) through enzyme expression, (2) the gut microbial dysbiosis provokes chronic inflammation through pattern recognition receptors to initiate DNA damage and cellular transformation, (3) the dysbiotic microbiota directly generates RONs to induce DNA damage and base excision repair (BER) intermediates, and (4) the release of bacterial genotoxins and metabolites causes chronic inflammation, which in turn promotes DNA damage [51].

Interventions with EGCG as an epigenetic active antioxidant may provide valuable impact in the therapy of metabolic syndrome. Bose et al. (2008) showed an effect of EGCG on HFD fed mice: the percentage of body fat and the visceral fat weight were reduced significantly (*p* ≤ 0.05) due to the supplementation of EGCG (3.2 g/kg diet) for 16 weeks when compared to control mice [11]. However, the bioavailability is low; intragastric administration of EGCG (75 mg/kg) resulted in 20.6 ng/g in the small intestine and 3.6 ng/g in colon. Oral administration of EGCG (equivalent with two or three cups of green tea) induced plasma levels of 0.2–0.3 *μ*M. It is regulated by the active efflux through the multidrug resistance-associate protein 2 on the apical surface of the intestine and the liver. The uptake predominantly takes place from enterocytes into the intestinal lumen and from the liver to the bile with excretion in the feces and little to none in the urine. EGCG and other tea catechins undergo extensive biotransformation: methylated by catechol-*O*-methyltransferase (COMT), glucuronidated by UDP-glucuronosyltransferases, and sulfated by sulfotransferases [54]. However, depending on polymerization degree, EGCG also influences gut microbiota composition: higher polymerization results in a higher bioavailability in the gut as the absorption in the small intestine is negligible in contrast to low degree of polymerization [55]. Methylated catechins, ring fission products (like valerolactone), and phenolic products further degraded to phenylacetic and phenylpropionic acids are indicative of gut microbial transformations. However, the biological activities of these metabolites are lower than the activities of their parent compounds [54]. On the other hand EGCG has valuable direct impact as antioxidant, but also as epigenetic active substance influencing histone modification and/or DNA methylation patterns [27].

### 4.1. EGCG Protected DNA Damage Caused by HFD in the Liver

EGCG supplementation caused a decrease of DNA migration in liver while no effects were found in the colon in the HFD group. In the CD + EGCG even increased DNA damage was found in the colon. The divergent effects in the two organs may be due to the effect that catechin is present in the colon in higher concentration as in the liver. It was shown earlier that high doses of EGCG cause formation of ROS, as a consequence DNA damage in human cells [56].

As mentioned above, comets reflect single and double strand breaks as well as apurinic sites; our findings are indicative for a protective effect of the green tea catechin toward these types of lesions. Our findings are in partial agreement with results published by Kager et al. (2010) who gave EGCG orally to normal weight mice (as in the present experiment). The authors showed no evidence for a protective effect in standard SCGE experiments in colons and livers. However, a clear protective effect was seen in hepatocytes but not in colonocytes in this experiment in regard to formation of oxidized DNA bases [41]. The molecular mechanisms which account for the DNA-protective properties of EGCG involve scavenging of radicals that are typical for obesity [57] which has been found* in vivo* in rodents and also* in vitro* [58, 59] alternatively; indirect effects caused by activation of antioxidant enzyme via the transcription factors nuclear factor erythroid 2-related factor 2 (Nrf2) may play a role [60]

Oršolić et al. (2013) published results which were obtained with diabetic mice; they found even an increase of DNA migration in the liver after injection of EGCG for 7 days [61]. Notably, the authors used a relatively high dose of the catechin in this experiment (i.e., 50 mg/kg body weight) and it is known from* in vitro* studies that high concentrations of EGCG and other phenolics cause DNA damage as a consequence of radical formation [62, 63].

The patterns of gene expression of* Dnmt1* and* MLH1* which were observed in the liver are in partial agreement with results from the SCGE experiments; a decrease was observed in hepatocytes in obese animals compared to controls. However, EGCG did not compensate this effect but caused a further decline of the expression of both genes.

On the contrary a clear increase of* Dnmt1* was seen with the catechin in the colons of HFD animals while obesity itself had no impact on the transcriptional activity of both genes in colonocytes. It is well documented that the* MLH1* and* Dnmt1* play a key role in DNA repair processes, in particular mismatch repair [64, 65]. However, distinct differences which we found between the induction of DNA migration in SCGE experiments and decreased gene expression levels distract from the assumption of a direct relation between comet formation and repair processes which are controlled by* MLH1* and* Dnmt1*.

### 4.2. EGCG Decreased Inflammatory IL-6 and* MLH1* Gene Expression Reflected by Higher* MLH1* Promoter Methylation

In colon a significantly lower* IL-6* gene expression was induced by EGCG but did not affect significantly* MLH1* gene expression. In liver EGCG reduced* MLH1* gene expression in both diet groups. The mean methylation was higher in CD + EGCG compared to CD whereas in HFD + EGCG a decrease was shown in comparison to HFD. However, methylation status varies CpG specific and additionally diet specific.

EGCG also reduced the expression levels of TNF-*α*, IL-6, IL-18, and IL-1*β* mRNAs, the serum levels of TNF-*α*, and the activation of Stat3 and JNK proteins in diethylnitrosamine- (DEN-) induced liver tumor genesis treated C57BL/KsJ-*db/db* (*db/db*) obese mice [13], but also IL-6 synthesis in rat adjuvant-induced arthritis by administration of 100 mg/kg EGCG, intraperitoneally daily [66]. Ahmed et al. (2008) showed an increase in the synthesis of soluble gp130 protein, an endogenous inhibitor of IL-6 signaling and transsignaling [66]. Additionally EGCG induced a concentration and time dependent reversal hypermethylation of tumor suppressor genes such as* p16, RAR, MGMT*, and* MLH1* genes in human esophageal cancer cells [12].

### 4.3. EGCG Increased* Dnmt1* DNA Methylation Resulting in Tissue Specific Variances in Gene Expression

EGCG supplementation resulted in a significantly reduced gene expression of* Dnmt1* compared to, respectively, CD and HFD in the liver. In the colon EGCG compensates the decrease in gene expression due to HFD and results equalized to CD and with three times higher gene expression of* Dnmt1* compared to HFD. The methylation status in the promoter region of* Dnmt1* was higher in supplemented groups compared to both control groups (CD, HFD). A significant increase was shown in CpG1 and CpG3 in the liver. In colon CpG2 and CpG4 were affected, respectively, in HFD + EGCG or in CD + EGCG.

EGCG was already shown to be the most efficacious inhibitor of enzymatic DNA methylation* in vitro* in comparison to other tea polyphenols (catechin, epicatechin) and bioflavonoids (quercetin, fisetin, and myricetin). Inhibitory effects for SssI nmt- and* Dnmt1*-mediated DNA methylation were shown at a half maximal inhibitory concentration (IC50) of 0.21 and 0.47 *μ*M, respectively. Inhibitory mechanisms are mentioned on one hand via direct pathways and on the other hand indirect via* Dnmt*-mediated DNA methylation through increased formation of SAH, a potent inhibitor of S-adenosylmethionine- (SAM-) mediated reactions [67] and via altering the availability of methyl groups which are used to methylate catechol groups on polyphenols by catechol-O-methyltransferase [27]. Although Lee et al. (2005) mentioned rather an important influence of the presence of a physiologically relevant concentration of Mg2+ (such as 2 mM) on inhibitory potency of EGCG compared to a dependence on its own methylation [67]. EGCG is also suggested to induce Foxp3 promoter demethylation inducing differentiation and expansion of Treg via* Dnmt* inhibitory activity and to reduce T cell proliferation and cytokine production [68]. Inhibition of* Dnmt*s together with an inhibition of histone deacetylase is suggested to prevent the hypermethylation and the silencing of key genes [67]. Either gut microbial derived metabolites of EGCG, gallic acid (GA), and epigallocatechin (EGC) influence epigenetic gene expression via HAT inhibitors to a lesser extent [27].

### 4.4. EGCG Changed Obese Gut Microbial Profile to Lean Phenotype

HFD feeding caused a significant lower bacterial abundance in both HFD groups (HFD, HFD + EGCG) resulting in a lower microbial diversity compared to CD. The* Firmicutes/Bacteroidetes* ratio is significantly lower in HFD + EGCG but higher in CD + EGCG compared to corresponding control group. Main changes due to EGCG intervention are decreased* Lactobacilli* abundance and also a lower abundance of* F. prausnitzii* in HFD groups with highest abundance in CD.* Butyryl CoA: acetate CoA-transferase* gene significantly increased in CD + EGCG whereas in HFD + EGCG the* butyrate kinase* significantly increased.* Akkermansia* are reduced to be abundant with EGCG supplementation.

It is already known that HFD, Western lifestyle, impacts gut microbiota composition. Diet quality and quantity are important influencing factors on bacterial community composition and metabolic/immunological activity of the host gut microbiota. Microbiota-mediated genomic instability but also immunological disequilibrium may be reduced due to phytochemicals. However, they are generally poorly absorbed in the small intestine; thus the impact of the close contact with the gut microbiota affects health benefits attributed to natural compounds. Results from green tea polyphenols, EGCG, give evidence to have a positive influence on gut microbiota composition. Unno et al. (2014) show not only changes in body and stool composition due to EGCG treatment but also changes in gut microbiota composition. However, these changes were dependent on dosage: 0.3% EGCG supplementation induced* Bifidobacterium*,* Lactobacillales*, and* Bacteroides* but reduced the abundance of* Clostridium* clusters. A concentration of 0.6% EGCG supplementation increased the abundance of* Lactobacillales *and* Bacteroides* but nearly depleted the abundance of* Clostridium* clusters and* Bifidobacterium* [26]. However, Unno et al. (2014) used Wistar rats and fed a commercial chow; thus comparison of both projects in gut microbiota composition, especially* Lactobacilli,* may be impaired on induced metabolic syndrome due to HFD feeding. However, we showed also an increase in* Bacteroidetes* and a decrease in* Clostridium* clusters impairing butyrate metabolism. Unno et al. (2014) showed lower levels of acetic and butyric acid but little influence on propionic acid due to 0.6% EGCG supplementation [26]. We even showed differences in butyrate formation pathways. The butyrate kinase pathway is more related to a Western diet, reflected in HFD fed mice (main fat resource: lard). Meanwhile the* butyryl CoA: acetate CoA-transferase* gene is associated with vegetarian feeding and to a lesser content available in omnivores [69].

In addition to EGCG as an epigenetically active antioxidant we also supplemented C57BL/6J mice with vitamin E (4.5 mg/kg b.w.) as a positive control which showed similar results compared to EGCG but with profound effects which will be published elsewhere. DNA damage showed a significant reduction in both organs in the HFD group but not in the CD group (tail intensity in CD-animals + vitamin E: 6.03 ± 0.95 in liver and 6.00 ± 1.13 in colon). The relative gene expression of* Dnmt1* in the colon increased with vitamin E supplementation in CD but significantly decreased in HFD. In all intervention groups a decreased gene expression was noted in the liver. The methylation status of* Dnmt1* in colon was lower. Furthermore, a positive correlation of* Dnmt1* mean methylation and DNA damage has been observed in liver whereas in CD a correlation has been found in the colon. Vitamin E supplementation affects specific CpG sites of* MLH1* inducing a lower gene expression of* MLH1* with HFD in liver and colon.

## 5. Conclusions

According to our results, EGCG might be suggested for the potential use for the prevention or in the therapy of obesity related and oxidative stress-induced health risks. One effect may derive from changes in GI microbiota and their anti-inflammatory effects by metabolites. Another effect may derive from antioxidative activities as well as epigenetic modifications observed on CpG methylation but also likely to include other elements of the epigenetic machinery. Interactions between antioxidative and epigenetic effects, for example, via ROS mediated breaks of* Dnmt* pathways, need to be explored.

## Figures and Tables

**Table 1:** Body weight and food and water intake of C57BL/6J male mice over a period of 4 months.

	Chow intake [g per day]				Water intake [ml per day]				Weight [g]			
	Month											
	1	2	3	4	1	2	3	4	1	2	3	4
Intervention												
CD	2.64 ± 0.17	2.11 ± 0.62	2.08 ± 0.61	1.99 ± 0.64	5.60 ± 0.99	5.36 ± 0.99	5.40 ± 0.91	5.05 ± 1.27	23.99 ± 1.50	25.92 ± 1.09	26.94 ± 1.42	27.88 ± 1.49
CD + EGCG	2.67 ± 0.12	2.61 ± 0.13	2.63 ± 0.12	2.62 ± 0.28	5.19 ± 0.61	5.13 ± 0.54	4.83 ± 0.65	4.60 ± 0.39	24.16 ± 1.52	25.81 ± 1.32	26.98 ± 1.53	28.43 ± 1.76
HFD	2.56 ± 0.10	2.59 ± 0.15	2.60 ± 0.13	2.56 ± 0.13	5.30 ± 0.43	5.13 ± 0.47	4.97 ± 0.54	5.07 ± 0.39	30.54 ± 3.43	37.62 ± 4.11	42.88 ± 4.58	45.99 ± 4.46
HFD + EGCG	2.57 ± 0.10	2.50 ± 0.10	2.58 ± 0.13	2.55 ± 0.16	5.07 ± 0.61	4.56 ± 0.40	4.43 ± 0.41	4.55 ± 0.31	30.74 ± 3.43	38.79 ± 3.07	44.72 ± 3.68	47.69 ± 3.45

**Table 2:** Sequence to analyze primers for CpG methylation analysis.

Gene	Primer	Sequence (5′-3′)	Size (bp)	GC%
*Dnmt1*	FW	Biotin - GTA GGT TGT AGA AGA TAG AAT AGT TTT GA	29	31
	RW	CCC ACT CTC TTA CCC TAT ATA ATA CAT	27	37
	Seq	CCC CTC CCA ATT AAT TTC	18	44.4

	*Sequence ID: gb|AH009208.2|*			
	*Dnmt1*: at reverse strand of chromosome 9: 20907205–20959888 (52684 bp).			

Sequence to analyze	**7104 – CGCGCGCGCG**AAAAAGC**CG**GGGTCT**CG**T - **7131**		27	7 CpGs

*MLH1*	FW	AGG GTA TTT TAG TTT TTA TTG GTT GGA GA	29	31
	RW	TTA CAC CTC AAT TCC TAA AAT CTC TAT CCC – Biotin	30	37
	Seq	TTT AGT TTT TAG AAA TGA GTT AAT A	25	16

	*Sequence ID: ref|XR_379849.3|*			
	*MLH1:* at reverse strand of chromosome 9: 111228228–111271786 (43559 bp)			

Sequence to analyze	19 - GAAGAG**CG**GAC**CG**TGAACTTTGA**CGCG**CAAG**CGCG **		64	8 CpGs
	TTGCCTTCTA-GCCTGGTGT**CG**GGC**CG**CTG - 82			

**Table 3:** Primers and TaqMan®-Probes targeting 16rRNA coding regions of bacteria.

Target organism	Primer/probe	Sequence (5′-3′)	Size (bp)	Conc. [pmol/*µ*L]	Reference
All *bacteria*	Fwd primer	ACT CCT ACG GGA GGC AG	468	10	[15]
	Rev primer	GAC TAC CAG GGT ATC TAA TCC		10	
	Probe	(6-FAM)-TGC CAG CAG CCG CGG TAA TAC-(BHQ-1)		2	

*Clostridium *cluster IV (Ruminococcaceae)	Fwd primer	GCA CAA GCA GTG GAG T	239	4	[16]
	Rev primer	CTT CCT CCG TTT TGT CAA		4	
	Probe	(6-FAM)-AGG GTT GCG CTC GTT-(BHQ-1)		2	

*Cluster XIVa (*Lachnospiraceae)	Fwd primer	GCA GTG GGG AAT ATT GCA	477	5	[16]
	Rev primer	CTT TGA GTT TCA TTC TTG CGA A		5	
	Probe	(6-FAM)-AAA TGA CGG TAC CTG ACT AA-(BHQ-1)		1,5	

*Bacteroidetes*	Fwd primer	GAG AGG AAG GTC CCC CAC	106	3	[17]
	Rev primer	CGC TAC TTG GCT GGT TCA G		3	
	Probe	(6-FAM)-CCA TTG ACC AAT ATT CCT CAC TGC TGC CT-(BHQ-1)		1	

*Bifidobacterium *spp.	Fwd primer	GCG TGC TTA ACA CAT GCA AGT C	125	3	[18]
	Rev primer	CAC CCG TTT CCA GGA GCT ATT		3	
	Probe	(6-FAM)-TCA CGC ATT ACT CAC CCG TTC GCC-(BHQ-1)		1.5	

**Table 4:** Primers (SYBR® Green) targeting 16rRNA coding regions of bacteria, *butyryl-coenzyme A (CoA) CoA transferase *genes, and *butyrate kinase* gene.

Target organism	Primer	Sequence (5′-3′)	Size (bp)	Conc. [pmol/*µ*L]	Reference
Lactobacilli	Fwd primer	AGC AGT SGG GAA TCT TCC A	352–700	4	[19]
	Rev primer	ATT YCA CCG CTA CAC ATG		4	

*Enterobacteria*	Fwd primer	AGC ACC GGC TAA CTC CGT	492–509	3	[20]
	Rev primer	GAA GCC ACG CCT CAA GGG CAC AA	834–856	3	

*Prevotella*	Fwd primer	CACCAAGGCGACGATCA	1458	2,5	[21]
	Rev primer	GGATAACGCCYGGACCT		2,5	

*Akkermansia*	Fwd primer	CAGCACGTGAAGGTGGGGAC	1505	2,5	[22]
	Rev primer	CCTTGCGGTTGGCTTCAGAT		2,5	

*BCoAT gene*	Fwd primer	GCIGAICATTTCACITGGAAYWSITGGCAYATG	~540	27	[23]
	Rev primer	CCTGCCTTTGCA ATRTCIACRAANGC		27	

*Butyrate kinase*	Fwd primer	TGCTGTWGTTGGWAGAGGYGGA	273	18	[24]
	Rev Primer	GCAACIGCYTTTTGATTTAATGCATGG		18	

**Table 5:** DNA methylation results, presented as relative methylation (mean ± SD) compared to CD or in the HFD groups, respectively, for every CpG (stars indicate significance: ^*∗*^ *p* value ≤ 0.05, ^*∗∗*^ *p* value ≤ 0.01).

Mean ± SD in %	CD + EGCG compared to CD	HFD compared to CD	HFD + EGCG compared to HFD
*Dnmt1* liver			
CpG1	1.71 ± 0.32^*∗*^	0.93 ± 0.25	1.62 ± 0.33^*∗*^
CpG2	1.14 ± 0.21	0.81 ± 0.12	1.10 ± 0.39
CpG3	1.37 ± 0.14^*∗∗*^	1.48 ± 0.73	1.18 ± 0.19
CpG4	1.19 ± 0.40	0.88 ± 0.48	1.39 ± 0.21
*Dnmt1* colon			
CpG1	1.07 ± 0.16	1.17 ± 0.24	0.89 ± 0.06
CpG2	1.15 ± 0.30	1.12 ± 0.14	1.19 ± 0.14^*∗*^
CpG3	0.98 ± 0.27	1.01 ± 0.19	1.23 ± 0.42
CpG4	1.28 ± 0.09^*∗*^	1.17 ± 0.12	0.98 ± 0.13
*MLH1* colon			
CpG1	0.38 ± 0.20^*∗*^	1.08 ± 0.46	0.98 ± 0.48
CpG2	0.66 ± 0.39	0.40 ± 0.03^*∗*^	0.65 ± 0.13^*∗*^
CpG3	1.04 ± 0.27	1.36 ± 0.44	0.87 ± 0.31
CpG4	0.62 ± 0.11^*∗*^	2.13 ± 0.12^*∗*^	0.76 ± 0.42
CpG5	1.75 ± 0.10^*∗*^	1.24 ± 0.09^*∗*^	0.55 ± 0.11^*∗*^
CpG6	0.37 ± 0.14^*∗*^	1.53 ± 0.37	0.68 ± 0.70^*∗*^
*MLH1* liver			
CpG1	0.56 ± 0.02^*∗∗*^	0.43 ± 0.03	0.98 ± 0.21
CpG2	1.65 ± 0.61	1.27 ± 0.37^*∗∗*^	1.80 ± 0.42^*∗∗*^
CpG3	1.11 ± 0.09	0.75 ± 0.13	1.16 ± 0.10
CpG4	2.73 ± 1.06^*∗∗*^	1.73 ± 0.08^*∗∗*^	1.13 ± 0.15
CpG5	1.20 ± 0.08^*∗∗*^	1.12 ± 0.26^*∗∗*^	0.86 ± 0.05^*∗∗*^
CpG6	1.20 ± 0.10^*∗*^	1.60 ± 0.47	0.68 ± 0.16^*∗∗*^

## Abbreviations

CD: Control diet
CD + EGCG: Control diet plus EGCG
cDNA: Complementary DNA
COMT: Catechol-*O*-methyltransferase
*Dnmt1*: DNA methyltransferase 1
DGGE: Denaturing gradient gel electrophoresis
EGC: Epigallocatechin
EGCG: (−)-Epigallocatechin-3-gallate
FFAs: Free fatty acids
GAPDH: Glyceraldehyde-3-phosphate-dehydrogenase
HAT: Histone acetyl transferase
HFD: High fat diet
HFD + EGCG: High fat diet plus EGCG
IC: Inhibitory concentration
IGF: Insulin-like growth factors
IL-6: Interleukin-6
MGMT: * O6-Methylguanine-deoxyribonucleic acid methyltransferase*
*MLH1*: MutL homologue 1
MMR: DNA mismatch repair
Nrf2: Nuclear factor erythroid 2–related factor 2
ROS: Reactive oxygen species
SAH: S-Adenosyl-l-homocysteine
SAM: S-Adenosylmethionine
SCFA: Short-chain fatty acids
SCGE: Single-cell gel electrophoresis
SD: Standard deviation
TNF-*α*: Tumor necrosis factor *α*.
